# Supplementary material for: Highly Efficient CRISPR/Cas9 Mediated Gene Editing in Ocimum basilicum ‘FT Italiko’ to Induce Resistance to Peronospora belbahrii
Source: Plants (Basel). 2023 Jun 21;12(13):2395. doi: 10.3390/plants12132395 (PMC10347046; doi:10.3390/plants12132395)
Supplement: Supplementary file 1 [file plants-12-02395-s001.zip › Captions of Supplementary materials.pdf]

Supplementary Materials

The following are available online at https://www.mdpi.com/article.....

S1 File: DNA Histograms of DAPI-stained nuclear preparation (Sysmex-Partec) from co-chopped leaves of diploid *O. sanctum* (as reference) and of *O. basilicum* FT ‘Italiko’.

S2 File: Protein alignment of ObDMR6 FT ‘Italiko’ and the 6 variants of ObDMR6 ‘Genoveser’ (Hasley et al., 2021); Identical amino acids are in black.

S3 File: Amino acid alignment of ObDMR6 ‘FT Italiko’ and AtDMR6. Identical and similar amino acids are in black and grey, respectively.

S4 File: WT: Nucleotide sequence and corresponding translation in protein sequence of WT (MT319764.1 [organism = Ocimum basilicum ‘FT Italiko’] Downy mildew resistance 6 (DMR6) mRNA, complete cds). The DMR6 characterizing motif WRDYLRL is highlighted in yellow; the NYYPPCP motif, responsible for binding the 2-oxoglutarate substrate, and the iron-binding HDH triplet, are highlighted in green (Pirrello et al., 2021). The domain that characterizes 2OG-F(II) oxygenase superfamily of oxidoreductase (pfam03171) is underlined, in amino acid sequence of WT). Nucleotide sequence and corresponding translation in protein sequence of edited plants (4A, 8A, 9B,11A, 14B, 17A2, 21D, 22B, 25B, 32A, 37A, 45B, 47C, 56A, HR5 and HR6). (The DMR6 characterizing motif WRDYLRL is highlighted in yellow. Mutation site is indicated with ★. Letters in light blue are divergent nucleotides in WT variants; letters in orange are base substitutions (bs); letters in red are modified aminoacids; letters in bold indicated the PAM sites).

S5 File: Genetic transformation of *Ocimum basilicum* 'FT Italiko' with *A. rhizogenes* (A) Induction of hairy roots (HRs) from callus; (B) Single and isolated HR representing an independent transgenic line.

S6 File: Alignment of *ObDMR6* WT natural variants. DNA fragment sequences of 15 WT individual clones. The divergent nucleotides are in yellow. The two overlapping sgRNA (sgRNA442 and sgRNA462) are in red. The PAM sites are conserved and are in red bold. Four different variants are indicated in grey (G/A), in light blue (G/T), in light green (A/A), and in light pink (A/T).

S7 File: Acclimatized T0 plant clones and T1 plants derived from self-pollination, for 22B and 21D lines.

S8 File: Statistical analysis of internode lengths (Student’s t-test) of fifteen T1 plants, arising from self-pollination of the T0 plants 21D and 22B, in comparison to WT (‘FT Italiko’ plants).
