# Supplementary material for: Highly Efficient CRISPR/Cas9 Mediated Gene Editing in Ocimum basilicum ‘FT Italiko’ to Induce Resistance to Peronospora belbahrii
Source: Plants (Basel). 2023 Jun 21;12(13):2395. doi: 10.3390/plants12132395 (PMC10347046; doi:10.3390/plants12132395)
Supplement: Supplementary file 1 [file plants-12-02395-s001.zip › S4 Rev 20 06 2023.pdf]

S4 file

WT: MT319764.1 [organism = *Ocimum basilicum* 'FT Italiko'] Downy mildew resistance 6 (DMR6) mRNA, complete cds; Nucleotide sequence and corresponding translation in protein sequence of WT. (The DMR6 characterizing motif WRDYLRL is highlighted in yellow; the NYYPPCP motif, responsible for binding the 2-oxoglutarate substrate, and the iron-binding HDH triplet, are highlighted in green (Pirrello et al., 2021). The domain that characterizes 2OG-F(II) oxygenase superfamily of oxidoreductase (pfam03171) is underlined, in amino acid sequence of WT).

1	ATG	GAA	CAT	AAG	GTG	ATT	AGT	GGA	ACA	CAG	TTC	AAA	AGC	CTT	CCC	45
1	M	E	H	K	V	I	S	G	T	Q	F	K	S	L	P	15
46	AGT	AGC	TAT	ATC	CGT	CCA	GAA	TCC	GAG	AGG	CCT	AAG	CTC	TCT	GAA	90
16	S	S	Y	I	R	P	E	S	E	R	P	K	L	S	E	30
91	GTT	GCT	GAT	TGC	AAT	AAT	GTT	CCC	ATC	ATT	GAC	TTG	GGC	TGT	GAA	135
31	V	A	D	C	N	N	V	P	I	I	D	L	G	C	E	45
136	GAT	CGT	AGA	CTG	TTA	ATT	AAA	CAG	ATT	GCC	GAT	GCT	TGT	CAA	GAA	180
46	D	R	R	L	L	I	K	Q	I	A	D	A	C	Q	E	60
181	TAT	GGA	TTC	TTC	CAG	GTG	ATC	AAT	CAT	GCA	GTG	CCA	AAG	GAG	CTG	225
61	Y	G	F	F	Q	V	I	N	H	A	V	P	K	E	L	75
226	GTG	AAT	AAA	ATG	GTG	GAG	GTG	GCT	CAT	GAA	TTC	TTT	AGT	TTA	TCC	270
76	V	N	K	M	V	E	V	A	H	E	F	F	S	L	S	90
271	GTG	GAG	GAG	AAG	ATG	AAG	TTA	TAC	TCC	GAC	GAC	CCT	TCC	AAA	ACC	315
91	V	E	E	K	M	K	L	Y	S	D	D	P	S	K	T	105
316	ATG	CGT	CTG	TCA	ACG	AGT	TTT	AAT	GTG	AGA	AAA	GAG	ACT	GTT	CAC	360
106	M	R	L	S	T	S	F	N	V	R	K	E	T	V	H	120
361	AAT	TGG	AGG	GAT	TAT	CTC	AGG	CTT	CAT	TGC	TAC	CCC	TTG	GAG	AAT	405
121	N	W	R	D	Y	L	R	L	H	C	Y	P	L	E	N	135
406	TAT	GTT	CCT	GAA	TGG	CCT	TCT	AAT	CCC	TCT	TCT	TTC	AAG	GAT	ATT	450
136	Y	V	P	E	W	P	S	N	P	S	S	F	K	D	I	150
451	GTA	AGT	AAC	TAC	TGT	AAA	GAA	GTT	CGA	GCC	TTT	GGA	TTC	AGA	TTG	495
151	V	S	N	Y	C	K	E	V	R	A	F	G	F	R	L	165
496	CAA	GAA	GCC	ATA	TCA	GAG	AGC	CTA	GGT	CTA	CAA	AAA	GAC	TCT	CTA	540
166	Q	E	A	I	S	E	S	L	G	L	Q	K	D	S	L	180
541	AAG	AAT	GTG	TTG	GGA	GAG	CAA	GGG	CAG	CAT	ATG	GCC	ATC	AAC	TAT	585
181	K	N	V	L	G	E	Q	G	Q	H	M	A	I	N	Y	195
586	TAT	CCT	GCA	TGC	CCA	CAA	CCT	GGT	CTC	ACG	TAT	GGA	TTA	CCT	GCA	630
196	Y	P	A	C	P	Q	P	G	L	T	Y	G	L	P	A	210
631	CAT	ACA	GAT	CCG	AAC	ACG	CTC	ACC	ATT	CTC	CTT	CAA	GAT	TTG	CAA	675
211	H	T	D	P	N	T	L	T	I	L	L	Q	D	L	Q	225
676	GTT	TCA	GGC	CTT	CAA	GTT	CTT	AAG	GAT	GAT	AAG	TGG	TTA	GCA	ATA	720
226	V	S	G	L	Q	V	L	K	D	D	K	W	L	A	I	240
721	AAA	CCT	CAT	CCA	GAT	GCT	TTT	GTC	ATC	AAT	ATT	GGT	GAC	CAA	ATC	765
241	K	P	H	P	D	A	F	V	I	N	I	G	D	Q	I	255
766	CAG	GCA	TTG	AGC	AAT	GGG	AAA	TAT	AGA	AGC	GTG	TGG	CAT	CGA	GCA	810
256	Q	A	L	S	N	G	K	Y	R	S	V	W	H	R	A	270
811	GTT	GTG	AAT	GCA	GAT	AAA	GCT	AGA	CTG	TCA	ATA	GCT	TCG	TTC	CTG	855
271	V	V	N	A	D	K	A	R	L	S	I	A	S	F	L	285
856	TGC	CCA	TGC	GAT	GCA	GCA	AAT	ATT	AGT	GCT	CCA	AAG	GAA	CTT	ACA	900
286	C	P	C	D	A	A	N	I	S	A	P	K	E	L	T	300

```
901    AGT GGA GAT GAT GGA GCC ATT TAC AGA GAT TAC ACA TAC GCT GAG   945
301     S   G   D   D   G   A   I   Y   R   D   Y   T   Y   A   E    315

946    TAC TAC AAA AAG TTC TGG AGC AGG AAT CTG GAC CAG GAC CAC TGC   990
316     Y   Y   K   K   F   W   S   R   N   L   D   Q   D   H   C    330

991    CTA GAA CTG TTC AAG AAT   1008
331     L   E   L   F   K   N
```

Nucleotide sequence and corresponding translation in protein sequence of edited plants (4A, 8A, 9B,11A, 14B, 17A2, 21D, 22B, 25B, 32A, 37A, 45B, 47C, 56A, HR5 and HR6). (The DMR6 characterizing motif WRDYLRL is highlighted in yellow. Mutation site is indicated with ★. Letters in light blue are divergent nucleotides in WT variants; letters in orange are base substitutions (bs); letters in red are modified aminoacids; letters in bold indicated the PAM sites).

>4A (divergent nucleotide in WT variant)

```
3      GAA ACT GTT CAC AAT TGG AGG GAT TAC CTC AGG CTT CAT TGC TAC   47
0       E   T   V   H   N   W   R   D   Y   L   R   L   H   C   Y    14

48     CCC TTG GAG AAT TTT GTT CCT GAA TGG CCT TCT AAT CCA TCT TCA   92
15      P   L   E   N   F   V   P   E   W   P   S   N   P   S   S    29
```

>8A (-2)

```
3      GAA ACT GTT CAC AAT TGG AGG GAT TAC CTC AGG CTT CAT TGC TAC   47
0       E   T   V   H   N   W   R   D   Y   L   R   L   H   C   Y    14
                                ★★
48     CCC TTG GAG AAT TTT GTT TGA ATG GCC TTC TAA TCC ATC TTC ATT   92
15      P   L   E   N   F   V   *   M   A   F   *   S   I   F   I
```

>9B (-1)

```
3      GAA ACT GTT CAC AAT TGG AGG GAT TAC CTC AGG CTT CAT TGC TAC   47
0       E   T   V   H   N   W   R   D   Y   L   R   L   H   C   Y    14
                                 ★
48     CCC TTG GAG AAT TTT GTT CTG AAT GGC CTT CTA ATC CAT CTT CAT   92
15      P   L   E   N   F   V   L   N   G   L   L   I   H   L   H    29

93     TCA AGG ATA TTG TAA GTA ACT ACT GTA AAG AAG TTC GAG CCT TTG   137
30      S   R   I   L   *   V   T   T   V   K   K   F   E   P   L    44
```

>11A (-1)

```
3      GAA ACT GTT CAC AAT TGG AGG GAT TAC CTC AGG CTT CAT TGC TAC   47
0       E   T   V   H   N   W   R   D   Y   L   R   L   H   C   Y    14
                                 ★
48     CCC TTG GAG AAT TTT GGC CTG AAT GGC CTT CTA ATC CAT CTT CAT   92
15      P   L   E   N   F   G   L   N   G   L   L   I   H   L   H    29

93     TCA AGG ATA TTG TAA GTA ACT ACT GTA AAG AAG TTC GAG CCT TTG   137
30      S   R   I   L   *   V   T   T   V   K   K   F   E   P   L    44
```

>14B (bs)

```
3      GAA ACT GTT CAC AAT TGG AGG GAC TAT CTC AGG CTT CAT TGT TAC   47
0       E   T   V   H   N   W   R   D   Y   L   R   L   H   C   Y    14
```

```
48    CCC TTG GAG AAA TAC GCG CCT GAA TGG CCA TCT AAT CCC TCT TCA   92
15     P   L   E   K   Y   A   P   E   W   P   S   N   P   S   S    29
```

>17A2 (-1)

```
3     GAG ACT GTT CAC AAT TGG AGG GAT TAC CTC AGG CTT CAT TGC TAC   47
0      E   T   V   H   N   W   R   D   Y   L   R   L   H   C   Y    14
                               ★
48    CCC TTG GAG AAT TTT GTT CTG AAT GGC CTT CTA ATC CAT CTT CAT   92
15     P   L   E   N   F   V   L   N   G   L   L   I   H   L   H    29

93    TCA AGG ATA TTG TAA GTA ACT ACT GTA AAG AAG TTC GAG CCT TTG   137
30     S   R   I   L   *   V   T   T   V   K   K   F   E   P   L    44
```

>21D (bs)

```
3     GAG ACC GTA CAC AAC TGG AGA GAC TAT CTC AGG CTT CAT TGC TAC   47
0      E   T   V   H   N   W   R   D   Y   L   R   L   H   C   Y    14

48    CCC TTG GAG AAT TTT GCG CCT GAA TGG CCA TCT AAT CCC TCT TCA   92
15     P   L   E   N   F   A   P   E   W   P   S   N   P   S   S    29
```

>22B (-1)

```
3     GAG ACT GTT CAC AAT TGG AGG GAT TAC CTC AGG CTT CAT TGC TAC   47
0      E   T   V   H   N   W   R   D   Y   L   R   L   H   C   Y    14
                               ★
48    CCC TTG GAG AAT TTT GTT CTG AAT GGC CTT CTA ATC CAT CTT CAT   92
15     P   L   E   N   F   V   L   N   G   L   L   I   H   L   H    29

93    TCA AGG ATA TTG TAA GTA ACT ACT GTA AAG AAG TTC GAG CCT TTG   137

30     S   R   I   L   *   V   T   T   V   K   K   F   E   P   L    44
```

>25B (bs)

```
3     GAA ACT GTA CAC AAC TGG AGA GAC TAT CTC AGG CTT CAT TGT TAC   47
0      E   T   V   H   N   W   R   D   Y   L   R   L   H   C   Y    14

48    CCC TTG GAG AAA TAC GCG CCT GAA TGG CCA TCT AAT CCC TCT TCT   92
15     P   L   E   K   Y   A   P   E   W   P   S   N   P   S   S    29
```

>32 A (-1)

```
3     GAG ACC GTT CAC AAC TGG AGA GAC TAT CTC AGG CTT CAT TGT TAC   47
0      E   T   V   H   N   W   R   D   Y   L   R   L   H   C   Y    14
                           ★
48    CCC TTG GAG AAA TAC GCC CTG AAT GGC CAT CTA ATC CCT CTT CTT   92
15     P   L   E   K   Y   A   L   N   G   H   L   I   P   L   L

93    TCA AGG ATA TTG TAA GTA ACT ACT GTA AAG AAG TTC GAG CCT TTG   137
30     S   R   I   L   *   V   T   T   V   K   K   F   E   P   L    44
```

>37 A (bs)

```
3     GAG ACC GTA CAC AAC TGG AGA GAC TAT CTC AGG CTT CAT TGT TAC   47
0      E   T   V   H   N   W   R   D   Y   L   R   L   H   C   Y    14
```

```
48     CCC TTG GAG AAA TAC GCG CCT GAA TGG CCA TCT AAT CCC TCT TCT   92
15      P   L   E   K   Y   A   P   E   W   P   S   N   P   S   S    29
```

>45B (+1)

```
3      GAA ACT GTT CAC AAT TGG AGG GAT TAC CTC AGG CTT CAT TGC TAC   47
0       E   T   V   H   N   W   R   D   Y   L   R   L   H   C   Y    14

48     CCC TTG GAG AAT TTT GTT CCT TGA ATG GCC TTC TAA TCC ATC TTC   92
15      P   L   E   N   F   V   P   *   M   A   F   *   S   I   F    29

93     ATT   95
30      I
```

>47C (+2 and bs)

```
3      GAG ACC GCA CAC AAC TGG GGA GAC TAT CTC AGG CTT CAT TGC TAC   47
0       E   T   A   H   N   W   G   D   Y   L   R   L   H   C   Y    14

48     CCC TTG GAG AAA GCA AGG TTC CTG AAT GGC CTT CTA ATC CAT GTT   92
15      P   L   E   K   A   R   F   L   N   G   L   L   I   H   V    29

93     CAT TCA AGT AAG CAA ACT CTT GTT TCA TTT AAT AAT GGA AGC AAA   137
30      H   S   S   K   Q   T   L   V   S   F   N   N   G   S   K    44

138    AGA ATG TAA CAA AAA ACA TCT TGC ATT TGC AGG GAT ATT GTA AGT   182
45      R   M   *   Q   K   T   S   C   I   C   R   D   I   V   S    59
```

>56A (-2)

```
3      GAG ACT GTT CAC AAT TGG AGG GAT TAC CTC AGG CTT CAT TGC TAC   47
0       E   T   V   H   N   W   R   D   Y   L   R   L   H   C   Y    14

48     CCC TTG GAG AAT TTT GTT TGA ATG GCC TTC TAA TCC ATC TTC ATT   92
15      P   L   E   N   F   V   *   M   A   F   *   S   I   F   I
```

>HR5 (-1)

```
3      GAA ACT GTT CAC AAT TGG AGG GAT TAC CTC AGG CTT CAT TGC TAC   47
0       E   T   V   H   N   W   R   D   Y   L   R   L   H   C   Y    14

48     CCC TTG GAG AAT TTT GTT CTG AAT GGC CTT CCT AAT CCA TCT TCA   92
15      P   L   E   N   F   V   L   N   G   L   P   N   P   S   S    29

93     TTC AAG TAA
30      F   K   *
```

>HR6 (-1)

```
3      GAA ACT GTT CAC AAT TGG AGG GAT TAC CTC AGG CTT CAT TGC TAC   47
0       E   T   V   H   N   W   R   D   Y   L   R   L   H   C   Y    14

48     CCC TTG GAG AAT TTT GTT CTG AAT GGC CTT CCT AAT CCA TCT TCA   92
15      P   L   E   N   F   V   L   N   G   L   P   N   P   S   S    29

93     TTC AAG TAA
```

30 F K *
