# Supplementary material for: Highly Efficient CRISPR/Cas9 Mediated Gene Editing in Ocimum basilicum ‘FT Italiko’ to Induce Resistance to Peronospora belbahrii
Source: Plants (Basel). 2023 Jun 21;12(13):2395. doi: 10.3390/plants12132395 (PMC10347046; doi:10.3390/plants12132395)
Supplement: Supplementary file 1 [file plants-12-02395-s001.zip › S5 .pptx]

## Slide 1
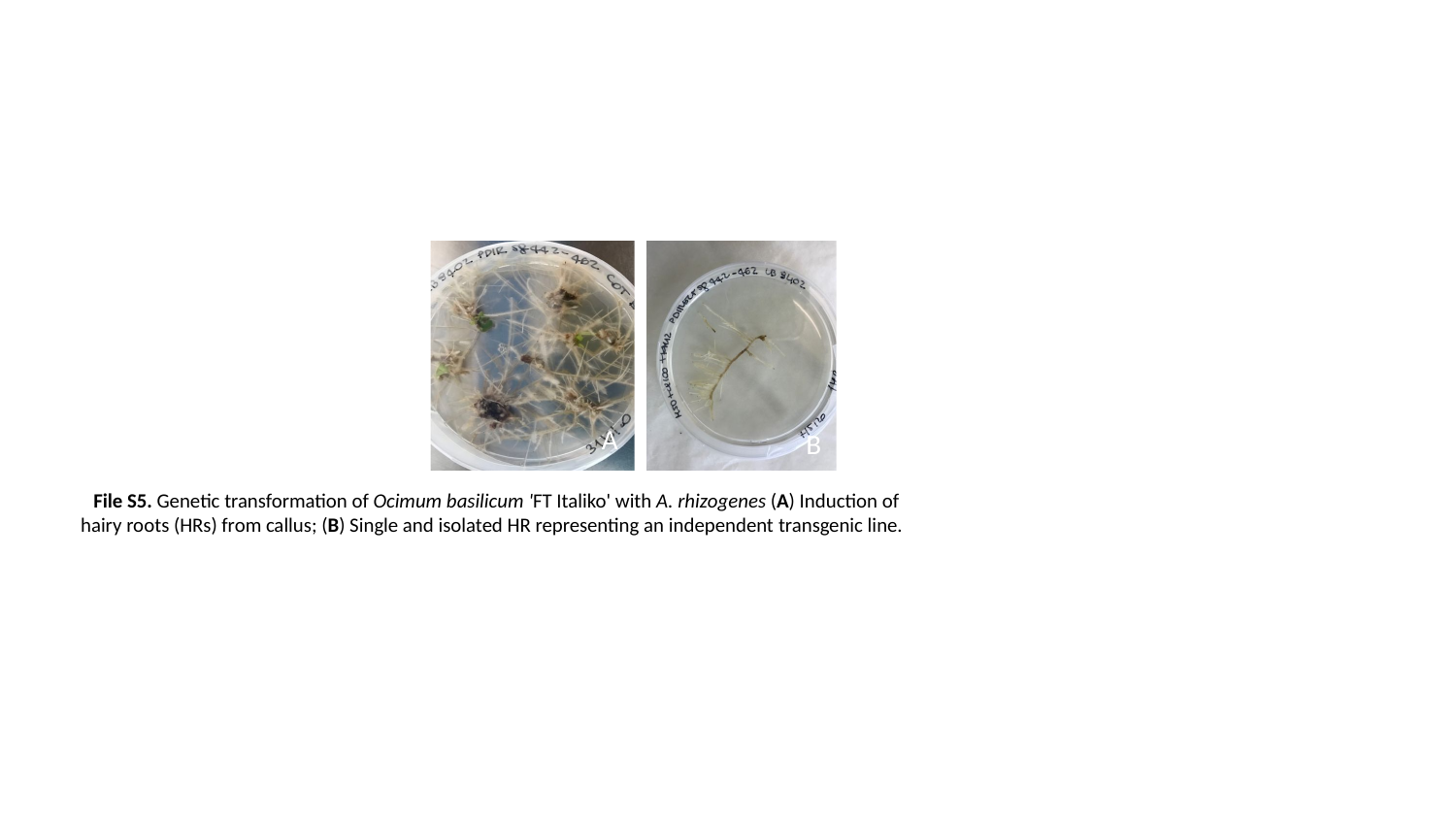

A
B
File S5. Genetic transformation of Ocimum basilicum 'FT Italiko' with A. rhizogenes (A) Induction of hairy roots (HRs) from callus; (B) Single and isolated HR representing an independent transgenic line.
