# Supplementary figures and images for: Highly Efficient CRISPR/Cas9 Mediated Gene Editing in Ocimum basilicum ‘FT Italiko’ to Induce Resistance to Peronospora belbahrii
Source: Plants (Basel). 2023 Jun 21;12(13):2395. doi: 10.3390/plants12132395 (PMC10347046; doi:10.3390/plants12132395)

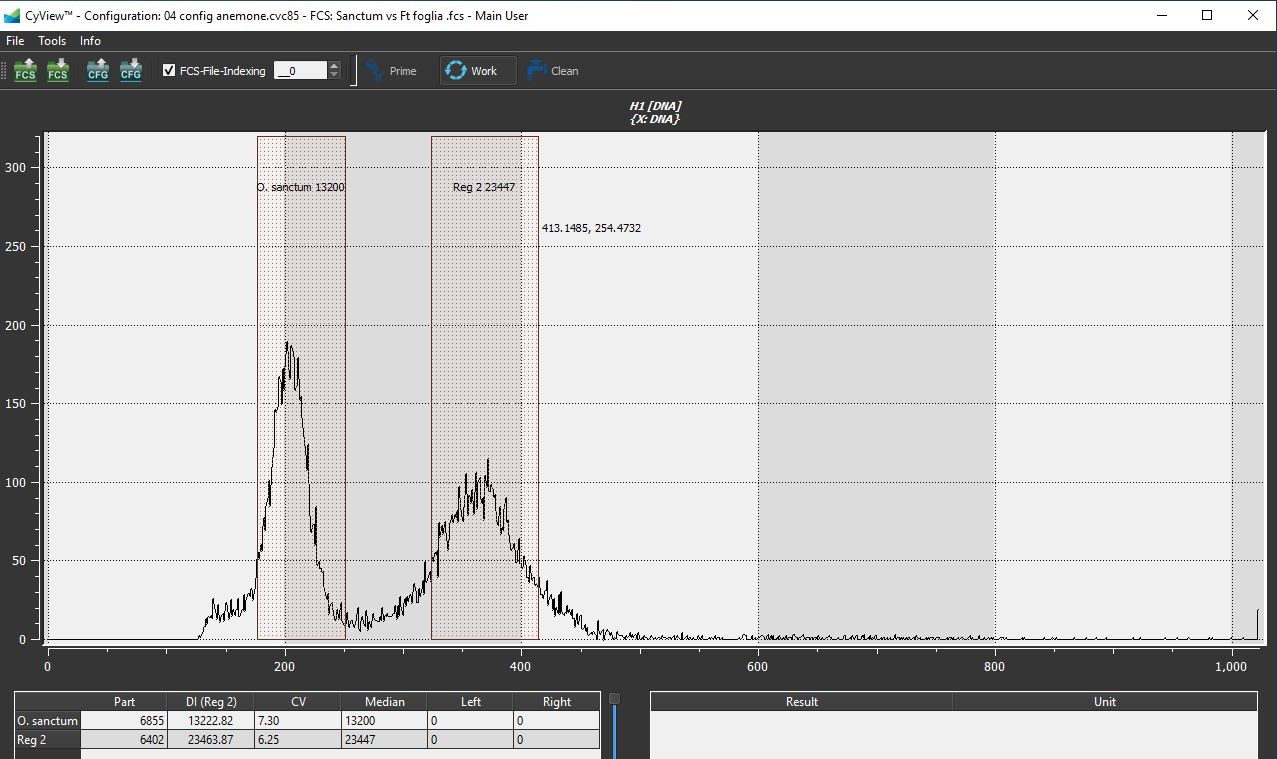

Supplement: Supplementary file 1 [file plants-12-02395-s001.zip › S1.jpg]

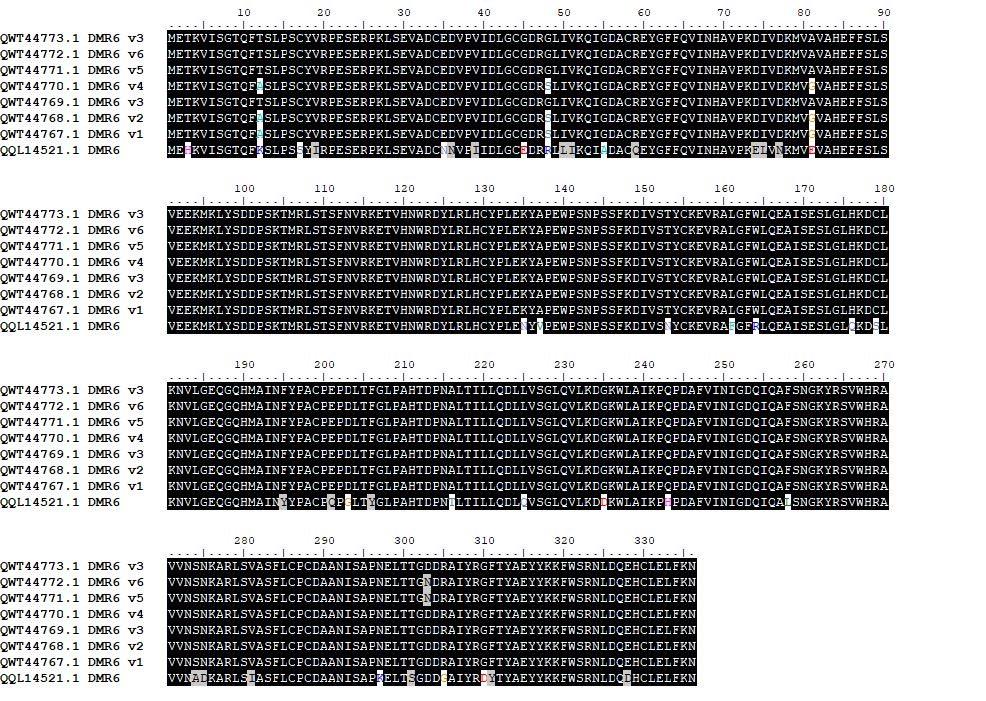

Supplement: Supplementary file 1 [file plants-12-02395-s001.zip › S2.JPG]

## Slide 1
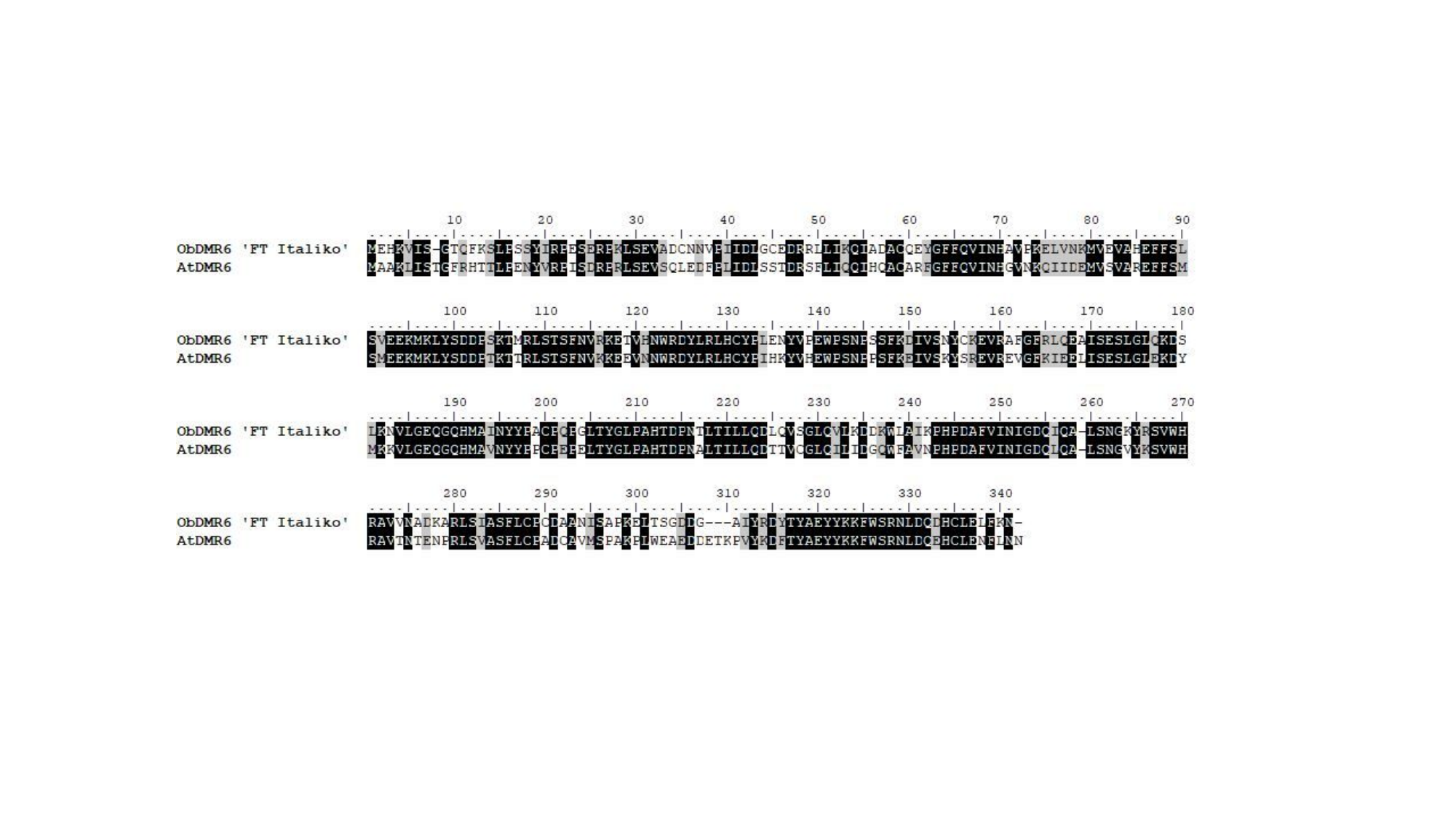

Supplement: Supplementary file 1 [file plants-12-02395-s001.zip › S3.pptx]

## Slide 1
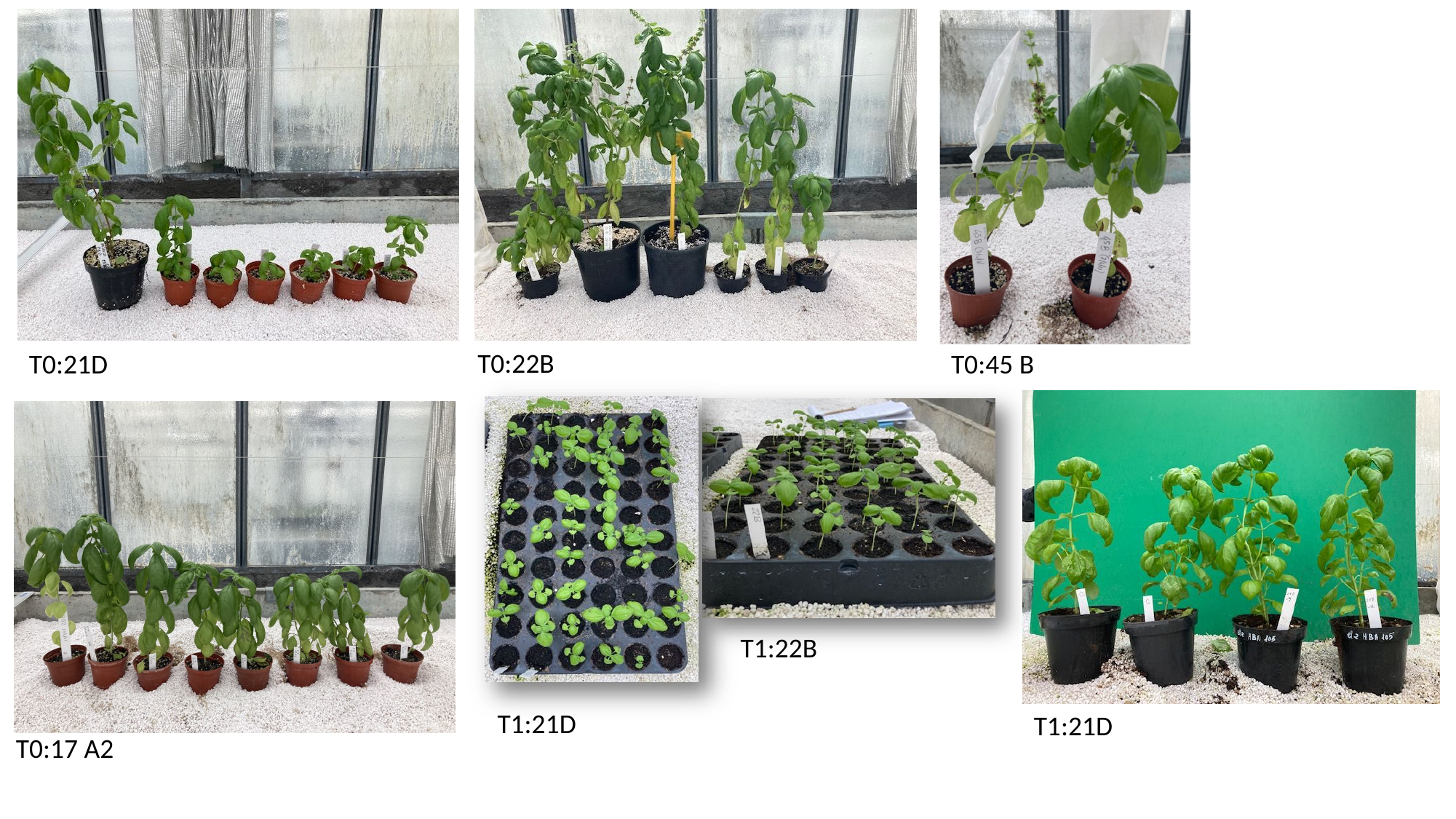

T0:22B
T0:45 B
T0:21D
T1:22B
T1:21D
T1:21D
T0:17 A2

Supplement: Supplementary file 1 [file plants-12-02395-s001.zip › S7.pptx]
